# Clinical distinctions in symptomatology and psychiatric comorbidities between misdiagnosed bipolar I and bipolar II disorder versus major depressive disorder

**DOI:** 10.1186/s12888-024-05810-3

**Published:** 2024-05-10

**Authors:** Zhiguo Wu, Jun Wang, Chen Zhang, Daihui Peng, David Mellor, Yanli Luo, Yiru Fang

**Affiliations:** 1 Department of Psychological Medicine, Renji Hospital, Shanghai Jiao Tong University School of Medicine, 160 Pujian Road, Shanghai, 200127 China; 2 Division of Mood Disorders, Shanghai Mental Health Center, Shanghai Jiao Tong University School of Medicine, Shanghai, China; 3 Shanghai Yangpu District Mental Health Center, Shanghai University of Medicine and Health Sciences, Shanghai, China; 4 School of Psychology, Deakin University, Melbourne, Australia; 5 Department of Psychiatry & Affective Disorders Center, Ruijin Hospital, Shanghai Jiao Tong University School of Medicine, 197 Ruijin 2nd Road, Shanghai, 200025 China; 6 Shanghai Key Laboratory of Psychotic Disorders, Shanghai, China; 7 CAS Center for Excellence in Brain Science and Intelligence Technology, Shanghai, China

**Keywords:** Major depressive disorder, Bipolar I disorder, Bipolar II disorder, Symptomatology, Psychiatric comorbidity

## Abstract

**Background:**

To explore the demographic and clinical features of current depressive episode that discriminate patients diagnosed with major depressive disorder (MDD) from those with bipolar I (BP-I) and bipolar II (BP-II) disorder who were misdiagnosed as having MDD .

**Methods:**

The Mini-International Neuropsychiatric Interview (MINI) assessment was performed to establish DSM-IV diagnoses of MDD, and BP-I and BP-II, previously being misdiagnosed as MDD. Demographics, depressive symptoms and psychiatric comorbidities were compared between 1463 patients with BP-I, BP-II and MDD from 8 psychiatric settings in mainland China. A multinomial logistic regression model was performed to assess clinical correlates of diagnoses.

**Results:**

A total of 14.5% of the enrolled patients initially diagnosed with MDD were eventually diagnosed with BP. Broad illness characteristics including younger age, higher prevalence of recurrence, concurrent dysthymia, suicidal attempts, agitation, psychotic features and psychiatric comorbidities, as well as lower prevalence of insomnia, weight loss and somatic symptoms were featured by patients with BP-I and/or BP-I, compared to those with MDD. Comparisons between BP-I and BP-II versus MDD indicated distinct symptom profiles and comorbidity patterns with more differences being observed between BP-II and MDD, than between BP-I and MDD .

**Conclusion:**

The results provide evidence of clinically distinguishing characteristics between misdiagnosed BP-I and BP- II versus MDD. The findings have implications for guiding more accurate diagnoses of bipolar disorders.

**Supplementary Information:**

The online version contains supplementary material available at 10.1186/s12888-024-05810-3.

## Background

Misdiagnosis of bipolar disorder (BP) is common in psychiatry clinics, with at least 20.8 − 61.5% of patients with BP being mistakenly diagnosed as having major depressive disorder (MDD) [1–4], which can lead to substantially guideline-disconcordant treatment for bipolar depression [5]. There are a variety of contributors to such misdiagnosis of BP as MDD. Firstly, the two disorders share extremely similar clinical phenomenology in the midst of depressive episodes [6], with identical diagnostic criteria applying to major depressive episodes and current diagnostic systems do not provide or elaborate meaningful differential features. Secondly, there is a lack of reliable and valid biomarkers that can be used to distinguish bipolar and unipolar depression. Finally, there is frequently a lack of assessment or an unsystematic assessment of current or past hypomania/mania of patients during their depressive episodes from the clinicians’ side and an unawareness of experiences of hypomania/mania from the patients’ side [1]. This is important as differential diagnosis between BP and MDD is solely based on determining the presence or absence of at least one lifetime occurrence of manic, hypomanic or mixed episode, meaning no assessment of the characteristics of depressive episodes is necessary in the differentiating procedures. However, depression rather than hypomania is typically dominant in BP [7, 8], which may further raise difficulties in identification of BP because most patients with BP-II present for treatment when depressed rather than hypomanic.

It is crucial then to explore clinical distinctions between BP and MDD during the depressive phases for the sake of diminishing misdiagnosis of BP. It has been reported in a few studies comparing BP-I and BP-II with MDD that patients with BP-I or BP-II were characterized by more likelihood of being male [2, 9], younger age at onset [2, 9], have had more depressive episodes [2, 9] and a greater family history of psychiatric disorders [2], in particular, mood disorders [9]. Highly replicated findings including a greater number of atypical [2, 10, 11], and psychotic symptoms [2, 11, 12] have been reported to differentiate BP-I from MDD, while patients with BP-II reported more mixed symptoms [12] and similar rates of psychotic symptoms as those with MDD [11]. Higher suicidality in BP-I and BP-II than in MDD has also been repeatedly reported [2, 12, 13].

Compared to classic and easily recognized BP-I, which is characterized by episodes of mania, BP-II is especially more difficult to differentiate from MDD. It is not surprising that a much higher proportion of patients with BP-II are misdiagnosed as having MDD than patients with BP-I (12.8% vs. 7.9%) [2]. Moreover, there are controversial ideas [14–16] about whether BP-II stands for a valid diagnostic category or just plays an intermediary role across the MDD-BP spectrum as a milder form of BP-I. On the one hand, it has been reported by head-to-head comparisons between the two bipolar subtypes that BP-II patients exhibit distinct and even more complex clinical phenotypes by presenting with an earlier onset of illness and a higher frequency of psychiatric comorbidities, depressive episodes and suicide attempts [17] than BP-I patients. On the other hand, other studies have reported fewer differences in depressive presentation between BP-II and MDD than between BP-I and MDD [9, 12, 18]. Conflicting results across studies fail to provide valid clinical correlates that can be used to distinguish BP-II from MDD.

China may have the largest population with BP in the world although a relatively low lifetime prevalence (with 0.6% in general, 0.4% of BP-I, 0.1% of BP-II and 0.1% of BP NOS) of BP has been reported [19]. Psychiatrists in this country are facing tremendous challenges in making accurate diagnoses of the illness due to a paucity of domestic research focusing on the phenomenology and diagnostic issues related to BP that may provide valid differentiating evidence [20]. The Previously reported proportion (20.8%) of patients with BP who were misdiagnosed as MDD in a nationwide survey is probably an underestimation of such misdiagnosis occurring in real clinical situations given the reality of low levels of mental health services and low quality of training for psychiatrists in China [21]. The above challenges, as well as the fact that 50.2% and 55.9% of the clinical population with BP have received guideline-disconcordant treatment during the depressive and maintenance phase [22, 23] respectively, highlight a great need for research on the differential diagnosis between BP and MDD.

The aim of this study was to explore the discriminating features of the current depressive episode of patients with either BP-I or BP-II who were misdiagnosed with MDD and patients with MDD in a clinical population drawn from 8 psychiatric settings in mainland China. In the present analysis, we focused on comparisons of depressive symptoms and psychiatric comorbidity between these three groups. These clinical variables are important potential distinguishers between BP and MDD depressive episodes, which were not fully examined in previously published comparable studies conducted in China [2, 3].

## Materials and methods

### Study settings and participants

Data used in the present analyses were derived from participants consecutively enrolled into the screening stage of *the Algorithm Guided Treatment Strategies for Major Depressive Disorder (AGTs-MDD)* [24, 25] which was conducted between June 2012 and May 2015 at 8 psychiatric facilities across mainland China. The study was approved by the Institutional Review Board of Shanghai Mental Health Center (IRB00002733-Shanghai Mental Health Center, China) and followed all relevant national and international ethical guidelines including the Declaration of Helsinki. Written informed consent was provided by each participant prior to study entry.

### Study design and measures

Patients, aged 18–75 years old, who were seeking outpatient or inpatient psychiatric care and currently experiencing depressive episodes were invited to participate in a two-stage screening interview. Those who had already had a diagnosis of bipolar disorder were excluded from the screening procedure according to the protocol of the AGTs-MDD study, which aimed to compare efficacy between two antidepressant treatment strategies (Algorithm Guided Treatment vs. Treatment as Usual) for patients with MDD [24]. In the first stage of the screening, each participant was interviewed by one research psychiatrist, who had worked at least 5 years in clinical practice. The interviewing process in this stage was routinely clinical, but the research psychiatrists were all trained for this project in how to make accurate differential diagnoses between MDD and BP possible. Patients who met DSM-IV TR diagnostic criteria for bipolar disorder as judged by the research psychiatrists were excluded and only those diagnosed with MDD at this stage entered into the next screening stage in which individual structured clinical interviews were conducted by well trained research assistants using the Chinese version Mini-International Neuropsychiatric Interview (the MINI) [26, 27] to determine their diagnoses. All the research assistants were psychiatrists with at least three years of practice in psychiatry. The participants who met DSM-IV TR diagnostic criteria for BP based on the results of MINI assessment were determined to be misdiagnosed BP cases and enrolled in the analysis along with the MDD cases. The MINI assessment has been proven to reliably establish DSM diagnoses of MDD, anxiety disorders, bipolar disorders, and so on [25, 27] and to identify BP-I and BP-II misdiagnosed as MDD in Chinese clinical populations [2]. No supplementary assessment tools (e.g., 32-item Hypomania Checklist or Mood Disorder Questionnaire) were employed in this study due to the need to reduce demand on participants.

Exclusion criteria included (i) an imminent risk for suicide or homicide; (ii) a severe and unstable general medical condition; (iii) being administered electroconvulsive therapy within 1 month prior to study entry; (iv) or for female patients, being pregnant or breastfeeding.

Other psychiatric diagnoses and measures used at the screening stage of this study have been detailed elsewhere [25]. In brief, diagnoses of anxiety disorders, recurrent depression, melancholic features, dysthymia, obsessive-compulsive disorder (OCD), post-traumatic stress disorder (PTSD), psychotic features, alcohol abuse, substance abuse, eating disorders and antisocial personality disorder were based on the MINI assessment. The interrater test reliability Cohen’s Kappa values for the above diagnoses were all > 0.87.

The determination of depressive symptoms was based on the 17-Item Hamilton Rating Scale for Depression (HRSD-17). For symptom frequency estimation, each of the HRSD-17 items was dichotomized as “absent” (score = 0) or “present” (scores = 1–4). We used low thresholds to indicate the presence of suicidal ideation and suicide attempt to underscore the importance of evaluation of suicide risk. Presence of suicidal ideation was determined by HRSD-17 item 3 (suicidality) score ≥ 1 or endorsement of any items of module C (suicidality) in the MINI. Presence of lifetime suicide attempt was determined by HRSD-17 item 3 (score = 4) or endorsement of item C6 (*In* your *lifetime, did you ever make a suicide attempt?*) of module C in the MINI. Other measures included gender and age at assessment.

### Statistical analyses

All statistical analyses were conducted with SPSS 21.0 (SPSS inc., Chicago, III). Summary statistics are presented as mean ± SD for continuous variables and percentages for categorical variables. To examine mean and proportional differences among the three groups, one-way ANOVAs and χ^2^ tests were used. The results were considered statistically significant at a two-tailed *p*<0.05, and a Bonferroni correction was used for multiple testing significance. A multinomial logistic regression analysis was performed to explore associations between clinical features and BP-I, BP-II and MDD diagnoses.

## Results

### Participants

A total of 1746 patents were prescreened. Of these, 65 patients did not finish the MINI assessment, 147 did not met criteria for a current major depressive episode and 71 had already been diagnosed as BP before the start of this study. This left 1463 participants who were included in the present study, among whom MDD, BP-I and BP-II accounted for 85.5% (*n* = 1251), 4.0% (*n* = 58) and 10.5% (*n* = 154), respectively. A total of 14.1% (*n* = 176) and 9.3% (*n* = 116) of MDD cases were taking antidepressants and combination medications. Specifically, 7 were taking mood stabilizers, 16 were taking antipsychotics, 83 were taking antianxietics, 21 were taking hypnotics, 10 were taking herbal medications and 8 were taking medications for physical illness. Only two BP-II cases were taking antidepressants and combination medications. None of BP-I cases was taking any medications.

### Demographics and depressive symptoms

Of the whole sample, 63.8% (*n* = 928) were female. Mean age at assessment was 37.21 years. The Mean total HRSD-17 score of participants was 20.78. As shown in Table 1, both participants with BP-I and with BP-II were younger than those with MDD (all *p* < 0.05, after Bonferroni correction). No significant differences between groups were detected in gender, melancholia features or illness severity as reflected by HRSD-17 total score. Cases with BP-II were more likely to be recurrent than those with MDD. Cases with BP-I and BP-II were both more likely to be characterized by concurrent dysthymia than those with MDD.

**Table 1:** Demographics and depressive symptoms

Clinical features	Total sample (*n* = 1463) *N* (%)	BP-I (*n* = 58) *N* (%)	BP-II (*n* = 154) *N* (%)	MDD (*n* = 1251) *N* (%)	χ^2^	*P*	BP-I vs. MDD			BP-II vs. MDD			BP-I vs. BP-II	
							OR ^a^	95% CI		OR ^b^	95% CI		OR ^a^	95% CI
Gender, female	928 (63.8)	30 (51.7)	87 (56.5)	811 (65.2)	8.34	0.015	0.57	0.34–0.97		0.69	0.49–0.97		0.82	0.45–1.51
Recurrence	647 (44.2)	31 (53.4)	90 (58.4)	526 (42.0)	17.03	0.000	1.58	0.93–2.68		1.94 ^*^	1.38–2.72		0.82	0.44–1.50
Dysthymia	220 (15.0)	16 (27.6)	35 (22.7)	169 (13.5)	16.56	0.000	2.44 ^*^	1.34–4.44		1.88 ^*^	1.25–2.84		1.30	0.65–2.58
Melancholia	990 (67.7)	33 (56.9)	101 (65.6)	856 (68.4)	3.71	0.157	0.61	0.36–1.04		0.88	0.62–1.25		0.69	0.37–1.28
Depressed mood	1450 (99.5)	57 (98.3)	152 (99.3)	1241 (99.6)	3.11	0.163	0.23	0.03-2.00		0.61	0.07–5.28		0.38	0.02–6.11
Guilt	1032 (70.9)	44 (75.9)	114 (74.5)	874 (70.2)	1.95	0.377	1.33	0.72–2.46		1.24	0.85–1.82		1.08	0.53–2.17
Suicidal ideation	986 (67.4)	40 (69.0)	103 (66.9)	843 (67.4)	0.08	0.959	1.08	0.61–1.91		0.98	0.68–1.40		1.10	0.56–2.10
Suicidal attempt (lifetime)	118 (8.1)	10 (17.2)	24 (15.6)	84 (6.7)	21.41	0.000	2.89 ^*^	1.41–5.92		2.56 ^*^	1.57–4.18		1.13	0.50–2.53
Initial insomnia	1033 (70.9)	37 (63.8)	89 (58.2)	907 (72.8)	15.60	0.000	0.66	0.38–1.14		0.52 ^*^	0.37–0.73		1.27	0.68–2.37
Middle insomnia	1153 (79.1)	45 (77.6)	104 (68.0)	1004 (80.6)	13.20	0.001	0.83	0.44–1.57		0.51 ^*^	0.35–0.74		1.63	0.81–3.30
Terminal insomnia	1051 (72.1)	39 (67.2)	86 (56.2)	926 (74.3)	22.95	0.000	0.71	0.40–1.24		0.44 ^*^	0.32–0.63		1.60	0.85–3.02
Interest loss	1397 (95.9)	54 (93.1)	148 (96.1)	1195 (95.9)	1.58	0.448	0.58	0.20–1.65		1.26	0.50–3.22		0.46	0.12–1.76
Retardation	1089 (74.7)	45 (77.6)	122 (79.7)	922 (74.0)	2.64	0.267	1.22	0.65–2.28		1.38	0.91–2.09		0.88	0.42–1.83
Agitation	812 (55.7)	29 (50.0)	105 (68.6)	678 (54.4)	11.96	0.003	0.84	0.45–1.42		1.83 ^*^	1.28–2.62		0.46 ^*^	0.25–0.85
Psychic anxiety	1365 (93.6)	54 (93.1)	145 (94.8)	1166 (93.4)	0.40	0.809	0.95	0.34–2.69		1.28	0.60–2.69		0.74	0.22–2.57
Somatic anxiety	1177 (80.7)	44 (75.9)	112 (73.2)	1021 (81.8)	7.37	0.025	0.70	0.34–1.30		0.61 ^*^	0.41–0.89		1.15	0.57–2.32
Gastrointestinal symptoms	929 (63.7)	32 (55.2)	83 (54.2)	814 (65.2)	8.99	0.011	0.66	0.39–1.12		0.63 ^*^	0.45–0.89		1.04	0.56–1.90
General somatic symptoms	1209 (82.9)	50 (86.2)	129 (84.3)	1030 (82.5)	0.78	0.677	1.32	0.62–2.83		1.14	0.72–1.80		1.16	0.49–2.76
Hyposexality	670 (45.9)	18 (31.0)	71 (46.4)	581 (46.6)	5.392	0.067	0.52	0.29–0.91		0.99	0.71–1.39		0.52	0.27–0.99
Hypchondrasis	679 (46.5)	29 (50.0)	69 (45.1)	581 (46.6)	0.41	0.816	1.15	0.68–1.94		0.94	0.67–1.32		1.22	0.66–2.23
Weight loss	649 (44.5)	12 (20.7)	44 (28.8)	593 (47.6)	33.48	0.000	0.29 ^*^	0.15–0.55		0.44 ^*^	0.31–0.64		0.65	0.31–1.34
Insight	735 (50.4)	21 (36.2)	74 (48.4)	640 (51.3)	5.32	0.070	0.54	0.31–0.93		0.89	0.64–1.24		0.61	0.32–1.13
Psychotic features	46 (3.1)	6 (10.3)	16 (10.5)	24 (1.9)	42.89	0.000	5.90 ^*^	2.31–5.05		5.97 ^*^	3.10-11.52		0.99	0.37–2.66
	Mean (SD)	Mean (SD)	Mean (SD)	Mean (SD)	*F*	*P*	*P*	95% CI		*P*	95% CI		*P*	95% CI
Age, years	37.21 (14.30)	33.00 (13.97)	33.47 (12.15)	37.86 (14.47)	8.98	0.000	0.013	1.05–8.67		0.000	1.99–6.78		0.831	-3.89-4.84
HRSD-17 total score	20.78 (5.45)	19.91 (5.59)	21.12 (6.04)	20.78 (5.37)	1.02	0.362	0.238	-0.58-2.32		0.475	-1.25-0.58		0.154	-0.45-2.86

Abbreviations: BP-I: patients with bipolar I disorder; BP-II: patients with bipolar II disorder; MDD: patients with majored depressive disorder; HRSD-17: the 17-Item Hamilton Rating Scale for Depression

^a^ ORs larger than 1.0 reflect higher prevalence in BP-I group

^b^ ORs larger than 1.0 reflect higher prevalence in BP-II group

^*^p<0.05 (after Bonferroni correction)

For depressive symptoms, the BP-I and BP-II groups showed only one difference, that being that BP-II cases were more likely than BP-I cases to report agitation. However, salient differences in depressive symptoms were found between BP-I and MDD cases and between BP-II and MDD cases. Specifically, both BP-I and BP-II cases were more likely than MDD cases to report a lifetime suicide attempt and psychotic features. BP-II cases were more likely than MDD cases to report agitation, but were less likely to report weight loss, initial, middle and terminal insomnia, somatic anxiety, and gastrointestinal symptoms. BP-I cases were more likely than MDD cases to report weight loss.

### Psychiatric comorbidities

As seen in Table 2, both of lifetime panic disorder and agoraphobia without panic co-occurred more frequently in BP-I and BP-II than in MDD. Panic disorder during current episode and antisocial personality disorder were also more common in BP-I than MDD cases. More salient differences in comorbidities between BP-II and MDD cases were found. BP-II cases were more likely than MDD cases to have comorbid social anxiety disorder, generalized anxiety disorder, OCD and eating disorders. A trend for higher levels of majority of psychiatric comorbidities except for panic disorder, OCD and antisocial personality disorder in BP-II than in BP-I cases appeared to exist although the differences were not significant after Bonferroni correction.

**Table 2:** Comorbidities

	Total sample (*n* = 1463) *N* (%)	BP-I (*n* = 58) *N* (%)	BP-II (*n* = 154) *N* (%)	MDD (*n* = 1251) *N* (%)	χχ^2^	*P*	BP-I vs. MDD			BP-II vs. MDD			BP-I vs. BP-II	
							OR ^a^	95% CI		OR ^b^	95% CI		OR ^a^	95% CI
Panic disorder (current)	31 (2.1)	4 (6.9)	5 (3.2)	22 (1.8)	7.12	0.019	4.14 ^*^	1.38–12.43		1.88	0.70–5.02		2.21	0.57–8.52
Panic disorder (lifetime)	55 (3.8)	5 (8.6)	12 (7.8)	38 (3.0)	12.51	0.002	3.01 ^*^	1.14–7.96		2.70 ^*^	1.38–5.28		1.12	0.38–3.32
Agoraphobia without panic	109 (7.5)	8 (13.8)	36 (23.4)	65 (5.2)	69.26	0.000	2.92 ^*^	1.33–6.41		5.57 ^*^	3.53–8.72		0.52	0.23–1.21
Social anxiety disorder	126 (8.6)	8 (13.8)	28 (18.2)	90 (7.2)	23.09	0.000	2.96	0.95–4.49		2.87 ^*^	1.81–4.55		0.72	0.31–1.69
Generalized anxiety disorder	277 (19.0)	12 (20.7)	46 (30.1)	219 (17.5)	14.08	0.001	1.23	0.64–2.36		2.02 ^*^	1.39–2.94		0.61	0.29–1.25
OCD	93 (6.4)	7 (12.1)	17 (11.0)	69 (5.5)	10.34	0.006	2.35	1.03–5.37		2.13 ^*^	1.22–3.72		1.11	0.43–2.82
PTSD	20 (1.4)	0 (0.0)	3 (1.9)	17 (1.4)	0.74	0.565	0.96	0.94–0.97		1.44	0.42–4.98		0.72	0.66–0.79
Alcohol abuse	20 (1.4)	1 (1.7)	4 (2.6)	15 (1.2)	2.63	0.185	1.45	0.19–11.14		2.20	0.72–6.71		0.66	0.07–6.01
Eating disorders	11 (0.8)	1 (1.7)	4 (2.6)	6 (0.5)	8.06	0.019	3.64	0.43–30.74		5.56 ^*^	1.55–19.33		0.65	0.07–5.97
Antisocial personality disorder	7 (0.5)	2 (3.4)	2 (1.3)	3 (0.2)	10.28	0.005	14.86 ^*^	2.43–90.70		5.51	0.91–33.24		2.70	0.37–19.60

Abbreviations: BP-I: patients with bipolar I disorder; BP-II: patients with bipolar II disorder; MDD: patients with majored depressive disorder; OCD: Obsessive-Compulsive Disorder; PTSD: Post-Traumatic Stress disorder

^a^ ORs larger than 1.0 reflect higher prevalence in BP-I group

^b^ ORs larger than 1.0 reflect higher prevalence in BP-II group

^*^p<0.05 (after Bonferroni correction)

### Results of multinomial logistic regression analysis

As shown in Table 3, results of multinomial logistic regression analysis using MDD as the reference category indicated BP-I and BP-II were both more likely to be associated with recurrence (OR = 1.90, 95% CI: 1.07–3.37 and OR = 2.07, 95% CI: 1.42–3.03, respectively), presence of antisocial personality disorder (OR = 11.53, 95% CI: 1.31– 101.29 and OR = 7.24, 95% CI: 1.01–51.79, respectively) and absence of weight loss (OR = 0.36, 95% CI: 0.17–0.74 and OR = 0.54, 95% CI: 0.35–0.83, respectively). BP-I was also more likely than MDD to be associated with lifetime suicide attempt (OR = 2.38, 95% CI: 1.07– 5.29) and comorbid current panic disorder (OR = 4.59, 95% CI: 1.16–18.20) and less likely to be associated with female gender (OR = 0.54, 95% CI: 0.30–0.96); whereas BP-II was more likely to be associated with lower level of illness severity based on HRSD-17 total score (OR = 0.93, 95% CI: 0.89– 0.98), presence of psychotic features (OR = 2.86, 95% CI: 1.32– 6.19), concurrent comorbid agoraphobia without panic (OR = 3.28, 95% CI: 1.96–5.50) and absence of terminal insomnia (OR = 0.60, 95% CI: 0.36–0.87) and somatic anxiety (OR = 0.62, 95% CI: 0.39–0.97). There were no significant differences in predictors that were associated with BP-I or BP-II when the two disorders were compared directly.

**Table 3:** Multinomial logistic regression model for factors associated with BP-I and BP-II versus MDD

	BP-I vs. MDD				BP-II vs. MDD				BP-I vs. BP-II		
	OR ^a^	95% CI	*P*		OR ^b^	95% CI	*P*		OR ^a^	95% CI	*P*
Age	1.02	0.99–1.04	0.116		1.01	0.99–1.03	0.157		1.01	0.98–1.04	0.561
HRSD-17 total score	0.98	0.92–1.06	0.650		0.93	0.89–0.98	0.003		1.05	0.97–1.14	0.211
Gender (Female)	0.54	0.30–0.96	0.035		0.78	0.54–1.14	0.205		0.69	0.36–1.32	0.262
Recurrence	1.90	1.07–3.37	0.028		2.07	1.42–3.03	0.000		0.92	0.48–1.76	0.794
Dysthymia	1.43	0.71–2.90	0.320		1.03	0.63–1.66	0.917		1.39	0.63–3.09	0.413
Suicidal attempt (lifetime)	2.38	1.07–5.29	0.033		1.58	0.91–2.76	0.103		1.50	0.62–3.63	0.366
Initial insomnia	0.78	0.40–1.52	0.464		0.69	0.45–1.07	0.100		1.12	0.53–2.40	0.762
Middle insomnia	1.08	0.51–2.30	0.831		0.70	0.44–1.11	0.130		1.55	0.66–3.56	0.302
Terminal insomnia	1.01	0.50–2.03	0.975		0.60	0.36–0.87	0.009		1.81	0.83–3.94	0.135
Agitation	0.78	0.43–1.41	0.405		1.38	0.92–2.08	0.118		0.56	0.28–1.11	0.097
Somatic anxiety	0.84	0.41–1.71	0.630		0.62	0.39–0.97	0.037		1.36	0.62–3.01	0.446
Gastrointestinal symptoms	0.90	0.48–1.66	0.725		0.82	0.55–1.23	0.344		1.09	0.54–2.18	0.809
Weight loss	0.36	0.17–0.74	0.005		0.54	0.35–0.83	0.005		0.66	0.29–1.50	0.325
Psychotic features	2.54	0.83–7.77	0.103		2.86	1.32–6.19	0.007		0.88	0.28–2.83	0.837
Panic disorder (current)	4.59	1.16–18.20	0.030		1.78	0.54–5.87	0.343		2.58	0.51–13.08	0.253
Agoraphobia without panic	1.88	0.79–4.48	0.154		3.28	1.96–5.50	0.000		0.57	0.23–1.43	0.232
Social anxiety disorder	1.02	0.41–2.54	0.958		1.35	0.79–2.31	0.270		0.76	0.29–2.01	0.577
Generalized anxiety disorder	0.68	0.32–1.45	0.320		1.14	0.73–1.77	0.562		0.60	0.26–1.36	0.221
OCD	1.34	0.52–3.46	0.552		1.08	0.56–2.06	0.816		1.24	0.43–3.56	0.694
Eating disorders	2.03	0.21–19.47	0.540		2.45	0.61–9.81	0.207		0.83	0.08–8.52	0.875
Antisocial personality disorder	11.53	1.31-101.29	0.027		7.24	1.01–51.79	0.048		1.59	0.18–14.22	0.677

Abbreviations: BP-I: patients with bipolar I disorder; BP-II: patients with bipolar II disorder; MDD: patients with majored depressive disorder; HRSD-17: the 17-Item Hamilton Rating Scale for Depressionr; OCD: Obsessive-Compulsive Disorder

^a^ ORs larger than 1.0 reflect higher prevalence in BP-I group

^b^ ORs larger than 1.0 reflect higher prevalence in BP-II group

^*^p<0.05

## Discussion

This study provided a broad evaluation of phenomenology focusing on depressive symptoms and psychiatric comorbidities in 1463 whose initial diagnoses were MDD in their current depressive episodes. We found that: (1) a substantial proportion of patients with BP-I (4.0%) or BP-II (10.5%) was misdiagnosed; (2) broad illness characteristics including younger age, higher prevalence of recurrence, concurrent dysthymia, suicide attempts, agitation, psychotic features and psychiatric comorbidities, as well as lower prevalence of insomnia, weight loss and somatic symptoms were featured by patients with BP-I and/or BP-II, compared to those with MDD; (3) comparisons of BP-I and BP-II versus MDD showed distinct symptom profiles and comorbidity patterns with more differences in clinical presentation being shown between BP-II and MDD. The results reported here mirror the clinical reality and challenges in differentiating BP from MDD, add to the growing literature about consistent distinctions in clinical presentation between BP and MDD patients during their current depressive episodes, and provide supportive evidence for BP-II as a valid bipolar disorder category.

The proportion of participants with BP misdiagnosed as MDD found in this study is close to that (20.8%) of a comparable clinical population in China [2]. Of particular note is that the research psychiatrists involved in this study were all well trained in diagnoses of BP and MDD and therefore well-placed to detect misdiagnoses. Reasons for the misdiagnosis of BP in China, and potentially elsewhere, include that the use of categorical diagnoses (‘bipolar’ versus ‘unipolar’) of illness limits the potential diagnosis of bipolar spectrum disorder, which has been conceptualized as not fully-fledged bipolar disorder but something in between unipolar and bipolar disorder [28]. Patients suffering from it are typically resistant to antidepressants but usually respond effectively to treatments for bipolar depression. A substantial proportion of MDD cases in our analysis had some signs and symptoms associated with treatment resistant depression and bipolar spectrum disorder including agitation, anxiety, suicide attempts, psychotic symptoms and comorbid anxiety disorders [28]. This alerts us to the possibility that many MDD cases may have a bipolar spectrum disorder not captured by the current categorical diagnosis. In other words, the rates of misdiagnosis of ‘bipolar illnesses’ within the context of bipolar spectrum disorder may be even higher than identified.

This misdiagnosis may indicate that this sample of bipolar patients is very difficult to identify or that there is something very different between them and those who are more readily distinguishable. Higher proportions of BP-I and BP-II cases than MDD cases with concurrent dysthymia were found in the present analysis. This is unexpected since brief depressive episodes, along with recurrent episodes, have been reported to be more characteristic of BP compared to MDD [12, 29]. The chronic depression in BP cases may possibly make it hard for them to remember previous manic/hypomanic episodes and bias psychiatrists away from adequately asking for prior histories of mania/hypomania. It is also possible that BP cases with concurrent dysthymia might be experiencing mixed features [12, 30], especially those patients with BP-II, who were more likely than MDD patients to be characterized by agitation which is highly suggestive of mixed features [11, 31]. This finding underscores the need for careful assessment of bipolar features for patients presenting with chronic depression and co-occurring agitation.

We found highly replicated external validating characteristics of BP depression compared to MDD, which have been previously reported either in a Chinese clinical population [2] or in other cultural populations [11, 12, 29, 32–35]. This suggests those clinical distinguishers might function similarly in differentiating bipolar and unipolar depression across cultural contexts. Specifically, recurrence [2, 11, 34, 35] and agitation [12, 32, 33] were more likely to be seen in the BP-II group and psychotic features were more likely to be observed in both of the BP groups [2, 35]. The finding of younger age at assessment of both BP-I and BP-II cases than MDD cases is also consistent with previous findings [2]. Whereas other studies have reported higher frequency of suicidal ideation among patients with BP-II [35] than among those with MDD, we did not observe significant differences between groups. However, we found both BP-I and BP-II cases more often had lifetime suicide attempts than MDD cases, confirming a considerable contribution of each of the two bipolar subtypes to suicidal behavior [36].

Despite not collecting data on atypical symptoms in this study, which have been shown to be robust external validating characteristics of BP [2, 11, 35], we did find their opposite, typical vegetative symptoms including early/middle/terminal insomnia and weight loss, were less likely to be reported by BP-II patients than MDD patients. Lower rates of weight loss were also observed in the BP-I group than in MDD group. Thus, our finding that typical vegetative symptoms were more strongly associated with MDD did not rule out associations between atypical vegetative symptoms and BP.

BP-II cases also had lower rates of somatic symptoms including somatic anxiety and gastrointestinal symptoms than MDD cases. Lower frequency of somatic symptoms involving muscular, respiratory and genitourinary discomforts in BP (BP-I and BP-II combined) than MDD has been previously reported [37]. Somatic symptoms, along with typical vegetative symptoms, have been repeatedly reported to be frequently presented by depressed patients in Chinese studies that have usually focused on unipolar depression [38, 39]. They have in fact often been considered socio-cultural hallmarks of this population [40, 41], countering a biological understanding. Our findings suggest a less cultural shaping phenomenology of BP than MDD, and therefore advancing a biological understanding of BP could be a way forward [20]. Further research with the inclusion of more somatic symptoms is needed to test whether patients with either subtype of BP are less physically vulnerable during depressive phases than those with MDD.

Regarding psychiatric comorbidities, each of the two groups of BP cases had significantly higher prevalence than MDD cases of the majority of the tested comorbidities. Specifically, all four forms of anxiety disorders, as well as OCD and eating disorders co-occurred more frequently in BP-II than in MDD, and three (current and lifetime panic disorders, agoraphobia without panic and antisocial personality disorder) of the nine tested comorbidities were more likely to be endorsed by BP-I cases than MDD cases. Overall, in contrast to the BP-I and MDD comparisons, there were more salient differences in comorbidity patterns between BP-II and MDD. This is consistent with previous findings by Angst et al. [42], but differs from other studies reporting either few differences in comorbidities between BP-I and BP-II versus MDD [12] or higher prevalence of comorbid anxiety disorders in BP-I than MDD and BP-II patients [9]. In addition to its high prevalence, comorbid anxiety in BP contributes to adverse illness course, poorer functioning and lower life quality and raises challenges in pharmacological treatment [43]. However, there has been no increase in research interest in this topic over the last two decades, and important issues such as the conceptualization, etiology of anxiety in BP and treatment (in particular, psychological therapy) for anxiety in patients with BP are still under-researched [43]. Future studies on this topic are needed.

In the multivariate logistic regression using MDD as the reference diagnosis, several predictors were significantly associated with BP diagnoses. Some associations (recurrence, antisocial personality disorder and absence of weight loss) were non-specific to BP subtypes and others were specific to BP-I (male gender, suicide attempt and panic disorder) or BP-II (less illness severity, psychotic features, concurrent agoraphobia without panic, and absence of terminal insomnia and somatic anxiety). Previously reported predictors of BP diagnoses based on multivariate regression analysis included more episodes being characteristic of BP-I [2, 12], psychotic features being characteristic of BP-I and BP-II [2] or BP-I [11, 12], mixed features being characteristic of BP-II [11, 12], anxiety disorders being characteristic of BP-I [9] or BP-II [42], and any personality disorder being characteristic of BP-I and BP-II [9], with a few of them being replicated in the present analyses. Despite findings from previous studies and ours using the multivariate regression models for discriminating between BP and MDD diagnoses, synthesis of findings is limited by the use of different definitions of variables, assessment instruments and comparison groups across studies.

### Strengths and limitations

The strengths of this study include the large sample size, broad inclusion criteria and minimal exclusion criteria, as well as use of a structured diagnostic interview applied by well trained psychiatrists to obtain a more accurate diagnostic picture. There are a number of limitations. First, in this post-hoc analysis based on data from screening stages of a published clinical trial, the study sample is not representative of the overall naturalistic clinical population as patients who had already had a diagnosis of bipolar disorder before the start of this study and those who met criteria for bipolar disorder as judged by research psychiatrists at the first screening stage were excluded. That said we could only enroll participants with undiagnosed BP who were actually misdiagnosed as MDD. However, misclassification bias in this analysis should be small according to the stringent diagnostic assessment and this may provide an opportunity for clinicians to better understand the facets of bipolar depression that are extremely difficult to differentiate from MDD. Second, a causal relationship between clinical features and diagnoses cannot be determined due to the cross-sectional nature of the study. Third, the sample size of the BP-I group was relative small and prevalence of some comorbid conditions was relatively low, which might lower the statistical power of comparisons. Fourth, information like age at onset, family history of mental illness, number of episodes, mixed features and response to antidepressants (e.g. treatment resistance and antidepressant-induced agitated, anxious, mixed, or manic/hypomanic state), which may be potential distinguishers between diagnoses were not collected. Finally, the potential influence of current treatment for patients who were not medication-naïve on clinical presentation cannot be ruled out. Furthermore, the fact that few patients with BP were taking psychotropic medications limited our ability to compare medication use between groups or to explore potential associations between medication use and diagnosis.

## Conclusion

Our results provide supportive evidence that symptom profiles and comorbidity patterns differ significantly between misdiagnosed bipolar I and bipolar II versus MDD, with more complex clinical differences existing between BP-II and MDD in contrast to the BP-I and MDD comparisons. This has implications for improving diagnostic decision making by identifying potential distinguishers of the two bipolar subtypes from MDD.

Given that our diagnoses were based on DSM-IV TR which have now been updated, future research based on the DSM-5 and/or ICD-11 could focus on: misdiagnosis or overdiagnosis of bipolar disorder in real-world clinical settings, implications of DSM-5/ICD-11 changes in diagnostic criteria for bipolar disorder on diagnostic prevalence of this illness, clinical presentation of depressive episodes of bipolar disorder and bipolar spectrum disorder compared to MDD, predictors of conversion from unipolar depression to bipolar disorder, strategies for improving accurate diagnosis of bipolar disorder in routine clinical practice, as well as the etiology and treatment for psychiatric comorbidities (e.g. anxiety disorders) in bipolar disorder.

### Electronic supplementary material

Below is the link to the electronic supplementary material.

Supplementary Material 1

## Data Availability

The datasets generated for this study are available on request to the corresponding author.

## Abbreviations

AGTs-MDD: The Algorithm Guided Treatment Strategies for Major Depressive Disorder
BP-I: Bipolar I disorder
BP-II: Bipolar II disorder
BP: Bipolar disorder
HRSD-17: The 17-Item Hamilton Rating Scale for Depression
MDD: Major depressive disorder
MINI: The Chinese version Mini-International Neuropsychiatric Interview
OCD: Obsessive-compulsive disorder
PTSD: Post-traumatic stress disorder
