# Supplementary material for: Clinical distinctions in symptomatology and psychiatric comorbidities between misdiagnosed bipolar I and bipolar II disorder versus major depressive disorder
Source: BMC Psychiatry. 2024 May 10;24:352. doi: 10.1186/s12888-024-05810-3 (PMC11088069; doi:10.1186/s12888-024-05810-3)
Supplement: Supplementary file 1 — Supplementary Material 1 [file 12888_2024_5810_MOESM1_ESM.docx]

**SUPPLEMENTARY MATERIALS**

**Clinical distinctions in symptomatology and psychiatric comorbidities between misdiagnosedbipolar I and bipolar II disorder versus major depressive disorder**

Zhiguo Wu ^1, 2, 3 † *^, Jun Wang ^3 †^, Chen Zhang ^2^, Daihui Peng ^2^, David Mellor ^4^, Yanli Luo ^1^, Yiru Fang ^2, 5, 6, 7 * *^

^1^ Department of Psychological Medicine, Renji Hospital, Shanghai Jiao Tong University School of Medicine, Shanghai, China

^2^ Division of Mood Disorders, Shanghai Mental Health Center, Shanghai Jiao Tong University School of Medicine, Shanghai, China

^3^ Shanghai Yangpu District Mental Health Center, Shanghai University of Medicine & Health Sciences, Shanghai, China

^4^ School of Psychology, Deakin University, Melbourne, Australia

^5^ Department of Psychiatry & Affective Disorders Center, Ruijin Hospital, Shanghai Jiao Tong University School of Medicine, Shanghai, China

^6^ Shanghai Key Laboratory of Psychotic Disorders, Shanghai, China

^7^ CAS Center for Excellence in Brain Science and Intelligence Technology, Shanghai, China

^†^ Zhiguo Wu and Jun Wang contributed equally to this work.

^*^ Correspondence to: Zhiguo Wu, Department of Psychological Medicine, Renji Hospital, Shanghai Jiao Tong University School of Medicine, 160 Pujian Road, Shanghai 200127, China. E-mail address: [zhiguo_wu@yeah.net](mailto:zhiguo_wu@yeah.net)

^* *^ Correspondence to: Yiru Fang, Department of Psychiatry & Affective Disorders Center, Ruijin Hospital, Shanghai Jiao Tong University School of Medicine, 197 Ruijin 2nd Road, Shanghai 200025, China. E-mail address: [yirufang@aliyun.com](mailto:yirufang@aliyun.com)

**SupplementaryTable 1** Summarized results of comparisons of demographics, depressive symptoms and comorbidities among groups

| Clinical features and comorbidities |  |
| --- | --- |
|  |  |
| Gender, female | BP-I < BP-II < MDD ^*^ |
| Recurrence | BP-II ^a^ > BP-I > MDD ^*^ |
| Dysthymia | BP-I ^a^ > BP-II ^a^ > MDD ^*^ |
| Melancholia | BP-I < BP-II < MDD |
| Depressed mood | BP-I < BP-II < MDD |
| Guilt | BP-I > BP-II > MDD |
| Suicidal ideation | BP-I > MDD > BP-II |
| Suicidal attempt (lifetime) | BP-I ^a^ > BP-II ^a^ > MDD ^*^ |
| Initial insomnia | BP-II ^a^ < BP-I < MDD ^*^ |
| Middle insomnia | BP-II ^a^ < BP-I < MDD ^*^ |
| Terminal insomnia | BP-II ^a^ < BP-I < MDD ^*^ |
| Interest loss | BP-I < MDD < BP-II |
| Retardation | BP-II > BP-I > MDD |
| Agitation | BP-II ^a, b^ > MDD > BP-I ^*^ |
| Psychic anxiety | BP-I < MDD < BP-II |
| Somatic anxiety | BP-II ^a^ < BP-I < MDD ^*^ |
| Gastrointestinal symptoms | BP-II ^a^ < BP-I < MDD ^*^ |
| General somatic symptoms | BP-I > BP-II > MDD |
| Hyposexality | BP-I < BP-II < MDD |
| Hypchondrasis | BP-I > MDD > BP-II |
| Weight loss | BP-I ^a^ < BP-II ^a^ < MDD ^*^ |
| Insight | BP-I < BP-II < MDD |
| Psychotic features | BP-II ^a^ > BP-I ^a^ > MDD ^*^ |
| Age, years | BP-I ^a^ < BP-II ^a^ < MDD ^*^ |
| HRSD-17 total score | BP-I < MDD < BP-II |
| Panic disorder (current) | BP-I ^a^ > BP-II > MDD ^*^ |
| Panic disorder (lifetime) | BP-I ^a^ > BP-II ^a^ > MDD ^*^ |
| Agoraphobia without panic | BP-II ^a^ > BP-I ^a^ > MDD ^*^ |
| Social anxiety disorder | BP-II ^a^ > BP-I > MDD ^*^ |
| Generalized anxiety disorder | BP-II ^a^ > BP-I > MDD ^*^ |
| OCD | BP-I ^a^ > BP-II ^a^ > MDD ^*^ |
| PTSD | BP-II > MDD > BP-I |
| Alcohol abuse | BP-II > BP-I > MDD |
| Eating disorders | BP-II ^a^ > BP-I > MDD ^*^ |
| Antisocial personality disorder | BP-I ^a^ > BP-II > MDD ^*^ |

Abbreviations: BP-I, patients with bipolar I disorder; BP-II, patients with bipolar II disorder; MDD, patients with majored depressive disorder; HRSD-17, the 17-Item Hamilton Rating Scale for Depression; OCD, Obsessive-Compulsive Disorder; PTSD, Post-Traumatic Stress disorder.

^*^ p＜0.05 (comparisons among the three groups).

^a^ p＜0.05 (compared to MDD group, after Bonferroni correction).

^b^ p＜0.05 (compared to BP-I group, after Bonferroni correction)

**Supplementary Table 2** Summarized results of multinomial logistic regression model for factors associated with BP-I and BP-II versus MDD

| Clinical variables | BP-I vs MDD |  | BP-II vs MDD |  | BP-I vs BP-II |
| --- | --- | --- | --- | --- | --- |
| Age | BP-I > MDD |  | BP-II > MDD ^*^ |  | BP-II > BP-I |
| HRSD-17 total score | BP-I < MDD |  | BP-II > MDD ^*^ |  | BP-II > BP-I |
| Gender (Female) | BP-I < MDD ^*^ |  | BP-II < MDD |  | BP-II > BP-I |
| Recurrence | BP-I > MDD ^*^ |  | BP-II > MDD ^*^ |  | BP-II > BP-I |
| Dysthymia | BP-I > MDD |  | BP-II > MDD |  | BP-I > BP-II |
| Suicidal attempt (lifetime) | BP-I > MDD ^*^ |  | BP-II > MDD |  | BP-I > BP-II |
| Initial insomnia | BP-I < MDD |  | BP-II < MDD |  | BP-I > BP-II |
| Middle insomnia | BP-I > MDD |  | BP-II < MDD |  | BP-I > BP-II |
| Terminal insomnia | BP-I > MDD |  | BP-II < MDD ^*^ |  | BP-I > BP-II |
| Agitation | BP-I < MDD |  | BP-II > MDD |  | BP-II > BP-I |
| Somatic anxiety | BP-I < MDD |  | BP-II < MDD ^*^ |  | BP-I > BP-II |
| Gastrointestinal symptoms | BP-I < MDD |  | BP-II < MDD |  | BP-I > BP-II |
| Weight loss | BP-I < MDD ^*^ |  | BP-II < MDD ^*^ |  | BP-II > BP-I |
| Psychotic features | BP-I > MDD |  | BP-II < MDD ^*^ |  | BP-II > BP-I |
| Panic disorder (current) | BP-I > MDD ^*^ |  | BP-II > MDD |  | BP-I > BP-II |
| Agoraphobia without panic | BP-I > MDD |  | BP-II > MDD ^*^ |  | BP-II > BP-I |
| Social anxiety disorder | BP-I > MDD |  | BP-II > MDD |  | BP-II > BP-I |
| Generalized anxiety disorder | BP-I < MDD |  | BP-II > MDD |  | BP-II > BP-I |
| OCD | BP-I > MDD |  | BP-II > MDD |  | BP-I > BP-II |
| Eating disorders | BP-I > MDD |  | BP-II > MDD |  | BP-II > BP-I |
| Antisocial personality disorder | BP-I > MDD ^*^ |  | BP-II > MDD ^*^ |  | BP-I > BP-II |

Abbreviations: BP-I, patients with bipolar I disorder; BP-II, patients with bipolar II disorder; MDD, patients with majored depressive disorder; HRSD-17, the 17-Item Hamilton Rating Scale for Depressionr ; OCD, Obsessive-Compulsive Disorder.

^*^ p＜0.05.
